# Endoscopic management of adult-type rhabdomyoma of the glottis: case report and review of the literature^☆^

**DOI:** 10.1016/j.bjorl.2015.04.008

**Published:** 2015-09-07

**Authors:** Filippo Carta, Sara Sionis, Clara Gerosa, Roberto Puxeddu

**Affiliations:** a Department of Otorhinolaryngology, University of Cagliari, School of Medicine, Azienda Ospedaliero-Universitaria, P.O. S Giovanni di Dio, Cagliari, Italy; b Department of Pathology, University of Cagliari, School of Medicine, Azienda Ospedaliero-Universitaria, P.O. S Giovanni di Dio, Cagliari, Italy

## Introduction

Rhabdomyomas are benign mesenchymal tumors composed of striated mature skeletal muscle cells, being no more than 2% of all striated muscle tumors,^1^ distinguished in cardiac and extracardiac subtypes. Cardiac rhabdomyomas occur generally in children and are considered hamartomatous lesions, often associated with phacomatoses, such as tuberous sclerosis,^1^, ^2^ and hamartomas of the kidney and other organs.^1^ Extracardiac rhabdomyomas are clinically and morphologically subdivided in three subtypes: the vaginal, fetal and adult variants. The vaginal-type is a rare tumor-like polypoid mass, found in the vagina and vulva of middle-aged women. The fetal-type, with the subordinated juvenile rhabdomyoma,^3^ is prevalent in head and neck areas in children. Adult extracardiac rhabdomyomas present generally as unifocal head and neck tumors in middle-aged patients,^4^, ^5^ multifocal in 14–26% of cases.^6^ Adult rhabdomyomas occur in the soft tissues of the head and neck up to 70–93% of cases,^1^ while glottic lesions are extremely rare, and only 22 cases have been reported up to now. With this article we report an additional case of glottic adult-type rhabdomyoma and review the pertinent literature, with two aims: (I) assess the standard of care of this pathology, to avoid inadequate treatment and (II) increase its knowledge among surgeons and pathologists.

## Case report

**Figure 1:**
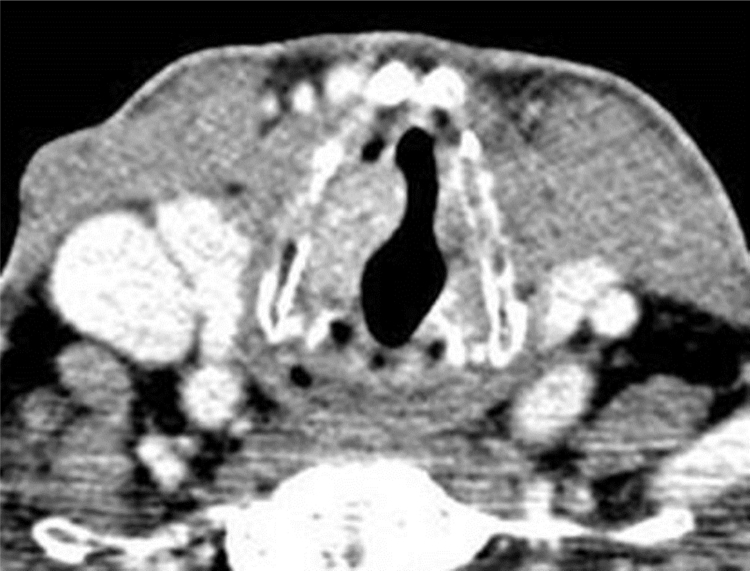
Contrast-enhanced computed tomography demonstrates an enhancing right laryngeal mass deeply located in the vocalis muscle.

**Figure 2:**
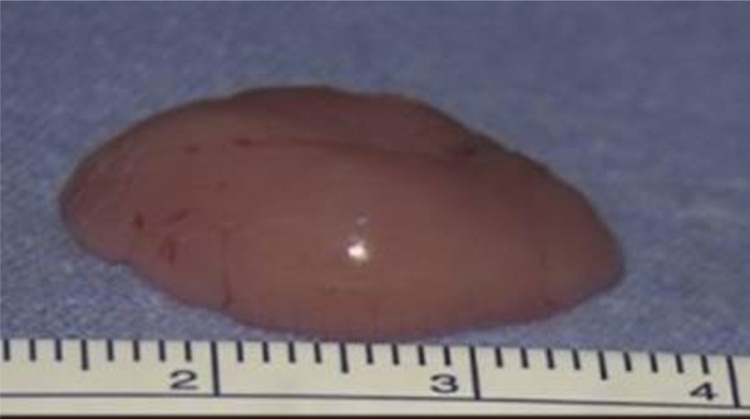
Laryngeal rhabdomyoma after excision: 22 mm × 15 mm × 9 mm.

**Figure 3:**
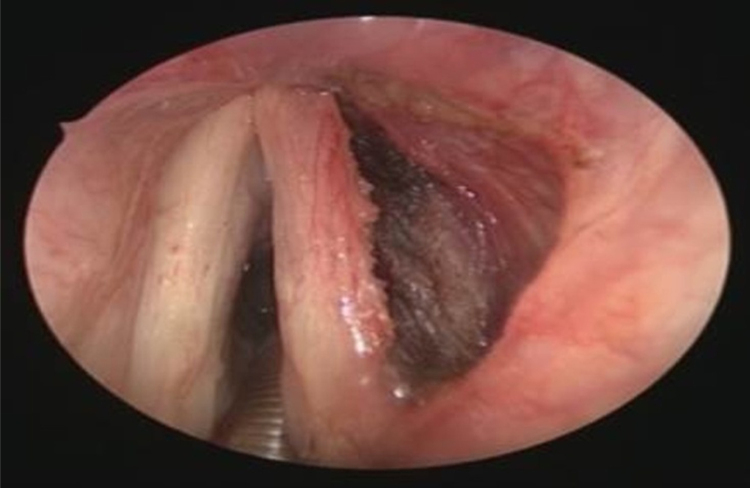
Endoscopic view after the removal.

**Figure 4:**
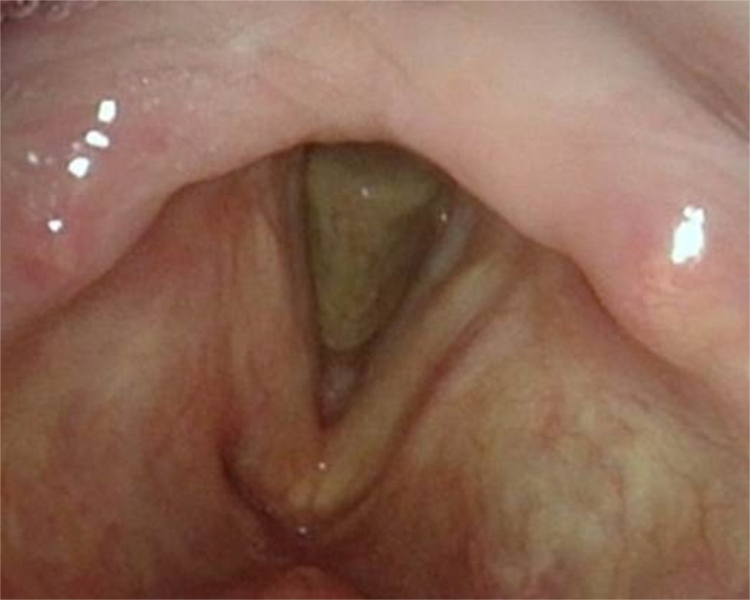
Indirect laryngoscopy at 12 months after surgery.

A 75-year-old male was referred to our department with a 4-year history of progressive dysphonia. Flexible scope examination showed a smooth submucosal swelling of the middle third of the right vocal cord, associated with impairment of vocal cord mobility. Contrast-enhanced computed tomography (CT) of the neck showed a deep right vocal cord lesion extended to the anterior paraglottic space, with low and uniform pathologic enhancement (Fig. 1). Clinical and radiological features suggested its benign nature and, therefore, conservative surgery was planned. The patient underwent transoral CO_2_ laser excision under general anesthesia with CO_2_ laser (Digital AcuBlade™, Lumenis™, Israel) set on 10 Watts, continuous wave in Super-Pulsed mode/emission, Acu-Blade 2 mm of length, under microscopic vision (focal length of 400 mm), through a microflap technique leaving the mucosa of the vocal cord intact. The tumor, deeply situated into the right vocal cord, was easily “en bloc” enucleated and appeared as an oval nodule of 22 mm in greatest dimension (Fig. 2). After the excision, the minus into the right thyro aritenoid muscle (Fig. 3) was left to heal by secondary intention. Postoperative course was uneventful: the patient was discharged 1 day after surgery and he regained normal vocal cord mobility and normal voice within 4 weeks. At histology, typical morphologic features of adult rhabdomyoma with sheets of large polygonal cells separated by few connective tissues were present. The cells had abundant eosinophilic cytoplasm with eccentrically placed nuclei, whereas in some areas cytoplasmic vacuolization with a centrally placed nucleus was found. Immunohistochemistry showed the cells to be strongly positive to skeletric muscle actin and desmin. At 12-month follow-up, the complete closure of the minus was observed (Fig. 4), with no evidence of recurrence.

## Discussion

Extracardiac adult and fetal types rhabdomyomas probably originate from skeletal muscle of the third and fourth branchial arches.^1^, ^7^ Their neoplastic nature was not clear because tumor cells usually do not express cell proliferation markers such as Ki-67 and PCNA, resembling more likely hamartomas than neoplasms.^7^ In 1992, Gibas and Miettinem demonstrated few chromosomal clonal anomalies supporting the neoplastic nature of rhabdomyomas.^8^ Before this case, 22 cases of adult-type laryngeal rhabdomyomas have been reported (Table 1): Johansen and coworkers, in 1995, reviewed all cases of adult rhabdomyomas of the larynx (*n* = 12) previously described^1^; after 1995, 10 further cases have been published. Age ranges from 16-year old to 79-year old (mean age 59 years, 59% of patients in the sixth and seventh decades, sex ratio M/F of 1:1.75); the tumor was found in the glottis in 12 cases, in the arytenoid in 4 patients and in the supraglottis in 7 patients; although stridor and airway obstruction can develop abruptly, the lesion generally remains asymptomatic, until it causes symptoms like dysphonia (86%), dysphagia (18%) and dyspnea (18%), that usually progress slowly (median duration-time of 2.5 years) (Table 1). Macroscopic appearance is usually a submucosal swelling with possible deep extension inside the laryngeal framework, but they may be sessile. Differential diagnoses include neurogenic or vascular tumors, oncocytoma, osteoma, Abrikossoff's tumor and rhabdomyomasarcoma.^1^ Radiographically adult rhabdomyoma presents as an homogenous lesion, isointense or slightly hyperintense to muscle on T1- as well as T2-weighted MRI and slightly hyperdense on CT.^4^ At histology, the adult and the fetal type have to be distinguished: the former closely mimics the structure of adult skeletal muscle and contains cells with PAS-positive granular or vacuolated cytoplasm, while the fetal type is composed with less differentiated neoplastic cells.^3^ Immunohistochemistry demonstrates the muscle immunophenotype, with strong positivity for muscle specific markers; in our case and in the literature, desmin appeared as a reliable marker.^1^, ^2^

**Table 1:** Adult-type laryngeal rhabdomyomas.

Source (year)	Location	Age/sex	Chief Complaint/duration of symptoms	Treatment	Comment
Clime et al. (1963)	Vocal cord	48/M	Hoarseness/3 months	Endoscopic excision	No recurrence reported
Battifora et al. (1969)	Glottis	55/M	Hoarseness/3 years	Excision with laryngofissure	No follow-up reported
Bianchi and Muratti (1975)	Right false vocal cord	52/F	Hoarseness	Endoscopic excision	No recurrence reported
Bagby et al. (1976)	Right false vocal cord	55/M	–	Endoscopic excision	No recurrence reported
Ebbesen et al. (1976)	Right ventricle	64/F	Hoarseness and foreign body sensation/6 months	Endoscopic excision	No recurrence reported
Winther (1976)	Vocal cord	39/M	Hoarseness/3 years	Endoscopic excision	Recurrence
Boedts and Mestdagh (1979)	Vocal cord	76/F	Hoarseness/2 months	Endoscopic excision	No recurrence reported
Kleinsasser and Glanz (1979)	Glottis	16/M	Acute airway obstruction/sudden onset	Total laryngectomy	Initial misdiagnosis of Rhabdomyosarcoma
Helliwell et al. (1988)	Left vocal cord	52/M	Hoarseness/6 months	Excision with lateral pharyngotomy	No recurrence reported
Heliwell et al. (1988)	Right vocal cord	66/M	Hoarseness/8 years	?	No follow-up reported
Hamper et al. (1989)	Arytenoid	51/F	Dyspnea and dysphagia	?	Recurrence
Johansen et al. (1992)	Left ventricule	51/M	Hoarseness, snoring/1 year	Hemilaryngectomy	No recurrence reported
Selme et al. (1994)	Vocal cord	31/F	Hoarseness	Complete removal after endoscopic biopsy	Clonal chromosomal anomalies
LaBagnara et al. (1999)	Vocal cord	69/F	Hoarseness/5 years	Endoscopic excision	Restauration of normal vocal cord function within 6 months
Orrit et al. (2000)	Arytenoid	66/M	Hoarseness and dysphagia/4 months	External removal	Vocal cord palsy
Brys et al. (2005)	Right false vocal cord	79/M	Hoarseness/5 years	External removal	Discharged after 10 days from the hospital
Liess et al. (2005)	Epiglottis	69/M	Asymptomatic	–	Multifocal
Jensen and Swartz (2006)	Right arytenoid	66/M	Dysphagia, hoarseness/3 years and sudden dyspnea	Endoscopic excision	Desmin high reactivity. 18 month of follow-up
Koutsimpelas et al. (2008)	Left aryepiglottic fold	72/F	Globulus and hoarseness/1 year	Endoscopic excision	Multifocal lesion
Farboud et al. (2009)	Arytenoid	76/M	Hoarsness, dysphagia and sleep-apnoea	Tracheostomy and endoscopic multiple debulking procedures	Bilateral
Friedman (2012)	Glottis	–	Dysphonia	Endoscopic excision	–
Cain et al. (2013)	Supraglottis	67/F	Hoarseness and progressive dyspnea	Tracheotomy and hemilaryngectomy	At 16 months complete glottic closure with phonation and no evidence of recurrence
Present case (2013)	Right vocal cord	75/M	Hoarsness/4 years	Endoscopic excision	No recurrence

Definitive treatment for laryngeal adult rhabdomyoma is complete excision; although extensive lesions reported in the literature required in 8 cases an external approach (Table 1), including a total laryngectomy, when glottic rhabdomyoma is confined to the endolarynx, the transoral approach should be preferred. Transoral minimally invasive laser CO_2_ assisted excision appears to be optimal in terms of efficacy and low morbidity: the vocalis muscle and the mucosa can be only incised without any removal. Since dedifferentiation of an adult rhabdomyoma to a malignant variety is not documented, a more invasive approach may appear an overtreatment, but a radical excision is mandatory since recurrences are possible (2 cases in the literature),^9^, ^10^ attributable to incomplete primary excision, that can occur since the consistence of the lesion is friable.

## Conclusion

Laryngeal rhabdomyoma is a rare benign tumor that has to be considered in the differential diagnosis of all submucosal laryngeal lesions. Conservative approach is advisable since the tumor can be endoscopically enucleated.

## Conflicts of interest

The authors declare no conflicts of interest.
